# Death of Dr. L. M. Kane

**Published:** 1850-02

**Authors:** 


We announce, with deep regret, the death of Dr. L. M. Kane, of Virginia, acting resident in the Pennsylvania Hospital. Although Dr. K. had been but a short time among us, he had won many friends by his quiet and amiable manners, and his warm devotion to the study of his profession. He was a graduate of the University of Virginia, and came to Philadelphia to study his profession practically, bearing with him the most flattering recommendations from those with whom he had been officially connected. He died of peritonitis on the 27th ult., after a short illness.

